# A mega-analysis of expression quantitative trait loci (eQTL) provides insight into the regulatory architecture of gene expression variation in liver

**DOI:** 10.1038/s41598-018-24219-z

**Published:** 2018-04-12

**Authors:** Tobias Strunz, Felix Grassmann, Javier Gayán, Satu Nahkuri, Debora Souza-Costa, Cyrille Maugeais, Sascha Fauser, Everson Nogoceke, Bernhard H. F. Weber

**Affiliations:** 1 Roche Innovation Center Basel, F. Hoffmann-La Roche Ltd, Basel, Switzerland; 2 Institute of Human Genetics, University of Regensburg, Regensburg, Germany

## Abstract

Genome-wide association studies (GWAS) have identified numerous genetic variants in the human genome associated with diseases and traits. Nevertheless, for most loci the causative variant is still unknown. Expression quantitative trait loci (eQTL) in disease relevant tissues is an excellent approach to correlate genetic association with gene expression. While liver is the primary site of gene transcription for two pathways relevant to age-related macular degeneration (AMD), namely the complement system and cholesterol metabolism, we explored the contribution of AMD associated variants to modulate liver gene expression. We extracted publicly available data and computed the largest eQTL data set for liver tissue to date. Genotypes and expression data from all studies underwent rigorous quality control. Subsequently, Matrix eQTL was used to identify significant local eQTL. In total, liver samples from 588 individuals revealed 202,489 significant eQTL variants affecting 1,959 genes (Q-Value < 0.001). In addition, a further 101 independent eQTL signals were identified in 93 of the 1,959 eQTL genes. Importantly, our results independently reinforce the notion that high density lipoprotein metabolism plays a role in AMD pathogenesis. Taken together, our study generated a first comprehensive map reflecting the genetic regulatory landscape of gene expression in liver.

## Introduction

Large genome-wide association studies (GWAS) have led to the identification of risk-associated variants with genome-wide significance for a multitude of diseases^1^. The very first successful GWAS identified an association between the complement factor H (*CFH*) locus on chromosome 1q31.3 and late stage age-related macular degeneration (AMD), the most common cause of blindness in industrialized countries^2^. The International AMD Genomics Consortium (IAMDGC) recently reported the most up-to-date list of genetic associations with 52 independent variants in 34 loci involved in AMD risk greatly extending our understanding of the genetic architecture of this blinding disease^3^. As one result, non-synonymous variants in five genomic loci point towards an involvement of the complement cascade as part of the innate immunity system^4–6^, implicating genes such as complement component 2 (*C2*), 3 (*C3*), 4 (*C4*), 9 (*C9*) as well as complement factor H (*CFH*), I (*CFI*), and B (*CFB*) in AMD pathology.

In addition, four AMD-associated loci harbour genes involved in high density lipoprotein (HDL) metabolism^7–9^. So far, the functional variants in the potential HDL-metabolism genes are not unambiguously identified, mainly due to extensive linkage disequilibrium between the strongest associated variants and other correlated variants regularly offering multiple plausible genes as disease-associated candidates. Although statistical methods can help to further reduce the number of candidate variants^10^, most of the signals associated with AMD are localized within non-coding regions of the genome^3^. These regions, however, may harbour sequences directly linked to gene expression such as 5′-prime untranslated regions or intronic sequences. On the other side, non-coding regions are often intergenic but nevertheless can have an effect such as recruiting transcription factors, which in turn can influence expression of nearby genes^11^. In general, such loci potentially harbour regulatory sequences in *cis* or *trans* to the gene regulated by the associated genetic variant.

Correlating the allele count at a variant locus and the expression of nearby genes in a given tissue can bridge the gap between the observed genetic association and understanding the mechanisms responsible for disease risk by defining an expression quantitative trait locus (eQTL)^12^. In recent years, thousands of eQTL were identified in multiple tissues by genome- and transcriptome-wide approaches^13^. Disease-associated genetic markers that represent a significant eQTL for a nearby gene can thus easily be identified. For AMD, so far only a single eQTL (rs79037040) affecting the expression of the tumor necrosis factor receptor superfamily, member 10a (*TNFRSF10A*) in white blood cells was reported to be associated with disease risk^14^. The lack of additional eQTL involved in AMD pathology can possibly be attributed to the observation that many eQTL studies are greatly underpowered^15,16^. In addition, although around 50% of known eQTL are common to several tissues^13^, many eQTL are likely to be specific for a given tissue or cell type.

The primary site of disease in AMD is the retinal tissue complex consisting of the retinal pigment epithelium (RPE), Bruch’s membrane and the choriocapillaris. The function of the liver is fundamentally different from the retina; thus the liver likely will react differently to environmental influences than retinal tissue. Furthermore, eQTL in liver might behave differently in retinal cells. However, it is challenging to sample a large number of human retinae and, as a consequence, no eQTL data from one of these cell types have been reported to date. Thus, we aimed at performing eQTL analysis in a surrogate tissue which expresses several genes of interest in loci associated with AMD, with the assumption that a polymorphism could have similar effects on gene expression in the surrogate tissue as in the retina. We selected liver as surrogate tissue since it is the main tissue for expression of genes of the complement system and of HDL metabolism. Moreover, gene products (e.g. proteins) of complement and of HDL metabolism expressed by the liver are frequently secreted into circulation where they exert various biological activities, and which could consequently influence AMD through its systemic effect in the choriocapillaris. With this rational we anticipated that investigating eQTL of these genes in liver could reveal important mechanistic insights into the association of these loci with AMD.

Several previous studies have published eQTL from liver tissue using different genotyping and expression profiling platforms^17–20^. Raw or curated data files of these studies are publicly available. In the present study, we have jointly analysed the data from the four independent liver eQTL resources by state-of-the-art methods, subsequent to rigorous quality control. In addition, the results were compared to published GWAS data for AMD risk variants. We show that a common, AMD associated deletion of the complement factor H related 1 and 3 genes (CFHR1/3) results in a markedly reduced expression of both genes in the liver. Furthermore, we show that two AMD risk variants are significant eQTL in liver affecting the expression of two genes involved in HDL metabolism.

## Results

### Data preparation

The main objective of this study was to identify significant cis-eQTL in liver tissue as part of our long-term goal to understand the functional consequences of genetic variants associated with complex diseases such as AMD. To this end, individual datasets publically available were merged although each one used distinct platforms to call genotypes and to measure gene expression (Table 1). Consequently, stringent quality control measures were applied to compile a data set of high quality genotypes and gene expression values comparable across studies. Altogether, the study comprised 6,256,941 imputed variants and expression values of 24,123 genes in 588 samples of European descent.

**Table 1:** Study and sample summary

Study	Schadt *et al*.^18^	Schroeder *et al*.^19^	Innocenti *et al*.^17^	GTEx Start/Mid^a^	Meta-analysis	Mega-analysis
Sample size before/after QC	178/178	149/149	208/178	97/83	588	588
Origin of liver tissue	Post-mortem tissue and resections from donor livers	Normal tissue resected during surgery for liver cancer	Post mortem tissue and resections from donor livers	Post mortem tissue	—	—
Transcriptome profiling platform	Agilent Custom 44k	Illumina Human WG-6v2.0	Agilent 4 × 44 k	RNA-seq (Illumina HiSeq2000)	—	—
Probes/genes before QC	40,638	48,701	45,015	56,318	—	—
Genes after QC	24,123	24,123	24,123	24,123	24,123	24,123
Genotyping platform	Affymetrix 500k; Illumina 650 Y	Illumina HumanHap300	Illumina 610 Quad	Illumina Omni 5 M/2.5 M^a^	—	—
Variants before QC	449,699	318,237	620,901	2,526,494/2,378,075^a^	—	—
Variants after QC	383,719	296,718	545,886	2,389,798/2,119,410^a^	—	—
Variants merged before imputation^b^	861,575	861,575	861,575	861,575	861,575	861,575
Variants after imputation and QC	6,256,941	6,256,941	6,256,941	6,256,941	6,256,941	6,256,941
eQTL variants (Q-Value < 1 × 10^−3^)	29,546	71,423	52,565	19,802	101,148	202,489
eQTL variants (Q-Value < 1 × 10^−3^, unique)	27,689	69,292	49,594	16,953	95,257	183,872
eQTL genes (Q-Value < 1 × 10^−3^, unique)	363	913	670	387	1,313	1,959
Overlapping eQTL genes with meta-analysis (Q-Value < 1 × 10^−3^)	215 (59.23%)	491 (53.78%)	408 (60.9%)	149 (38.5%)	1,313 (100%)	1,260 (64.32%)
Overlapping eQTL genes with mega-analysis (Q-Value < 1 × 10^−3^)	288 (79.34%)	688 (75.36%)	537 (80.15%)	207 (53.49%)	1,260 (95.96%)	1,959 (100%)
Independent signals (P-Value < 1 × 10^−6^)	—	—	—	—	—	2,060

QC = quality control; ^a^Omni 2.5 M for the first data release (GTEx start) and Omni 5 M for the mid-point release (GTEx mid). ^b^After quality control the genotype files of the four studies were merged into a single file and variants, which did not overlap in-between datasets, were assigned missing. We only kept variants which were genotyped in at least 100 samples.

### eQTL Analysis

First, we performed eQTL calculations for each of the four studies individually^13,17–19^. Local eQTL were calculated by including all variants on the same chromosome that are located within 1,000,000 base pairs (1 Mbp) up- or downstream of the transcription start site or polyadenylation site of a gene locus, respectively. Next, mixed effects models were used to perform a meta-analysis by including the effect sizes and standard errors obtained from each study separately. In order to account for multiple testing, we controlled the false discovery rate (FDR) to be smaller than 0.001^21^. At this threshold, 101,148 eQTL variants and 1,313 genes differentially regulated by the eQTL were identified (Table 1).

**Figure 1:**
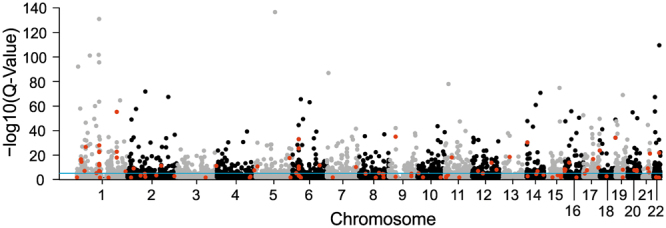
Manhattan plot of the eQTL mega-analysis in liver. A mega-analysis was conducted including 588 samples from four independent studies measuring eQTL variants in liver tissue. The Manhattan plot shows the −log_10_ Q-Values of the most significant variant for each of the 24,123 analysed autosomal genes. Additionally, 101 independent secondary signals were identified and are highlighted in red. The blue line depicts the threshold for significance at 1 × 10^−3^.

As meta-analysing data can result in a loss of statistical power^22–24^, we additionally performed a mega-analysis by directly estimating eQTL in the entire dataset comprising all four studies. The mega-analysis yielded 202,489 statistically significant eQTL variants affecting the expression of 1,959 genes while controlling the FDR to be less than 0.001 (Fig. 1, Table 1 and Supplementary Table S1). Compared to the results from the meta-analysis, the mega-analysis provided a two fold increase in the number of eQTL variants and a 1.5 fold increase in the number of differentially regulated genes. Of note, however, both mega- and meta-analysis discovered more significant results than any of the four individual studies alone (Table 1). Only 38.5 to 60.9% of the significant single study eQTL genes could be replicated in the meta-analysis. The GTEx study had the lowest replication rate, possibly due to its relatively small sample size (N = 83). The overlap of single study results and the mega-analysis is on average 19% higher (53.5 to 80.15%) than the overlap observed in the meta-analysis. As the mega-analysis reproduced 95.96% of the meta-analysis eQTL and detected many signals beyond, we decided to rely on the data of the mega-analysis for further calculations although this may represent a slight overestimation of eQTL derived from the available data set.

We next aimed to identify independent eQTL variants (independent hits) within a significant eQTL. Consequently, the eQTL analysis was repeated for each significant eQTL gene after additionally adjusting the linear regression model for the most significant variant identified for the eQTL gene. The procedure was reiterated until no additional significant variants were identified. In this analysis, a variant was regarded a significant independent eQTL for a given gene if the P-value associated with the regression slope was lower than 1 × 10^−6^. With this approach, we detected an additional 101 independent eQTL variants in 93 out of 1,959 liver eQTL genes (Fig. 1, Supplementary Tables S2 and S3). Of note, our analysis could not replicate the AMD associated eQTL rs797037040 previously shown to influence the expression of TNFRSF10A in blood^14^. This is owed to the fact that neither this variant nor any variant in linkage disequilibrium *(R* > *0.4*) *to* rs797037040 could be reliably imputed into the dataset.

### Characterization of eQTL-variants

**Figure 2:**
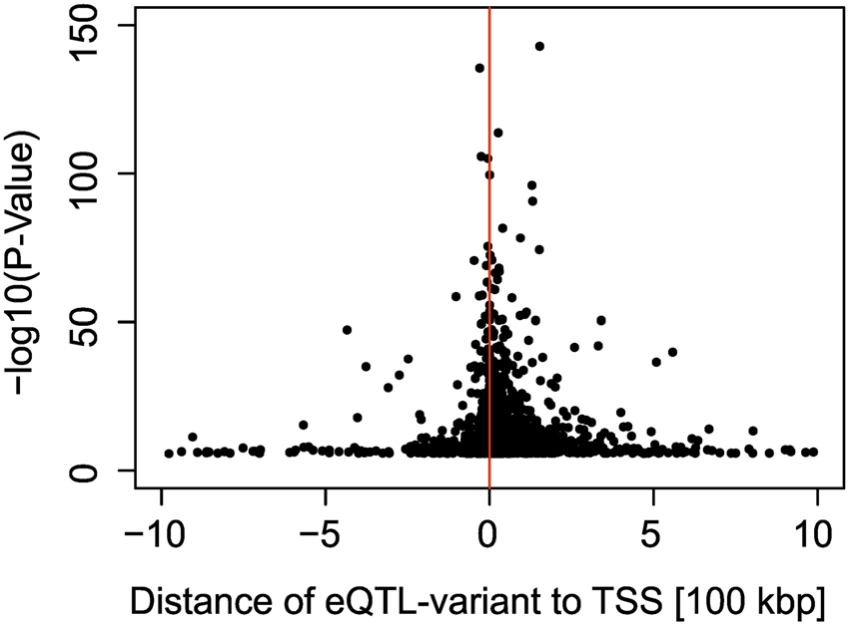
Characterisation of independent signal eQTL variants based on their genomic localisation. The distance to the nearest transcription start site (TSS) is plotted against the −log_10_ P-Values of the most significant variant at each eQTL gene, including secondary signals (independent hits). Negative/positive distances denote that the variant is located upstream/downstream of the TSS with regard to the direction of transcription.

We further localized all independent eQTL hits with regard to the transcription start site (TSS) of the affected gene (Fig. 2). We observed that the most significant eQTL variants were close to a respective TSS. Overall, 1,599 out of 2,060 (1,959 + 101) independent eQTL variants were within 100,000 base pairs of a nearest TSS, well in agreement with other studies^16,25–27^.

**Figure 3:**
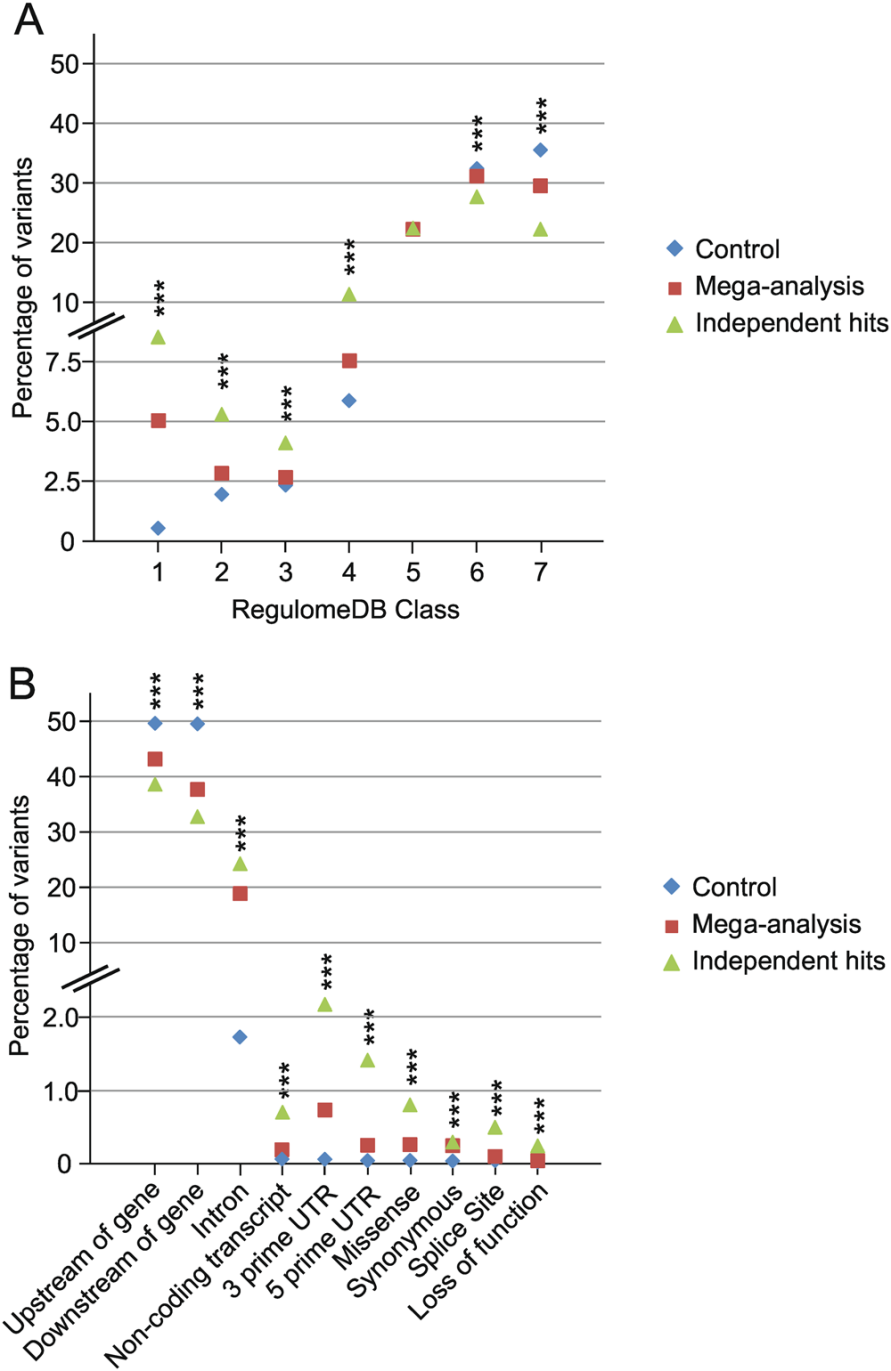
Functional annotations and predicted consequences of local eQTL-variants. Three sets of variants were evaluated by employing two different databases. Set one (control) includes random variants of the imputed genotype file, which are located next to at least one gene within a distance of a maximum of 1 Mb. Set two (mega-analysis) consists of all significant mega-analysis (Q-Value < 1 × 10^−3^) eQTL variants while the third group comprises the most significant variant of each independent hit (including the independent secondary signal variants). (**A**) The chart depicts the percentage of variants per variant set categorised into seven groups by RegulomeDB. The seven-level functional score is based on a synthesis of data derived from various sources: category 1 variants are very likely to affect binding and are linked to gene expression of a target gene (i.e. are known eQTL variants); categories 2 and 3 are likely to affect at least transcription factor binding and several other regulatory effects; categories 4–6 show minimal functional indication while category 7 variants lack evidence for any functional relevance. (**B**) The chart shows the percentage of variants classified into ten classes of consequences according to the Ensembl Variant Effect Predictor (VEP). For variant set two (mega-analysis) and three (independent hits) we only included the predicted consequence affecting the identified eQTL gene. For the control group, one random gene within a variant–gene distance of a maximum of 1 Mb was chosen. We selected the most severe effect, if the variant had different effects on transcripts of the same gene. ***P-Value for difference between groups <0.001.

We then evaluated the RegulomeDB^28^ scores of eQTL variants (Fig. 3A and Supplementary Table S4). As expected, eQTL variants (N = 183,872) were enriched in RegulomeDB classes one to four (P-values < 6.82 × 10^−09^), which represent variants with likely regulatory properties while categories 5 and higher show minimal to no functional relevance. In addition, eQTL variants with the smallest P-values and additional secondary signals (independent hits, N = 2,040) revealed an even stronger enrichment in classes one to four compared to controls and compared to all eQTL variants (P-values from 1.72 × 10^−04^ to 8.27 × 10^−11^).

To further characterise each eQTL signal for its most severe functional consequence relative to a known gene structure, we applied Ensembl *VEP*^29,30^ (Fig. 3B, Supplementary Table S5). Control variants were predominantly located upstream (49.22%) and downstream (49.09%) of known gene structures. Another 1.63% of the control variants were found in introns of genes. Less than 0.1% of the control variants were assigned to functional categories such as missense or untranslated transcript region (UTR). Interestingly, the proportion of intronic variants was significantly larger in both, the mega-analysis variants (19.72%, P < 1.00 × 10^−150^) and the independent hit variants (29.17%, P < 1.00 × 10^−150^) (Fig. 3B, Supplementary Table S5). Additionally, other predicted categories like UTR or coding region variants occurred more often (P-values < 1.72 × 10^−07^).

Taken together, our findings indicate that significant liver eQTL variants are more often localized within known gene structures and are likely regulatory variants as they are found within regions of transcription factor binding and open chromatin. In addition, the most significant variants are also the most likely functional variant in each eQTL. This is supported by findings that the most significant eQTL variants (i) show an increased level of enrichment in all relevant RegulomeDB score categories compared to all eQTL variants and (ii) are enriched within known gene structures such as introns or coding exons.

### Liver eQTL in AMD

Finally, we investigated whether any of the 52 independent AMD associated variants reported by Fritsche *et al*.^3^ coincides with the established liver eQTL. Out of 52 independent tag variants, only 31 variants had an allele frequency >5% and could be reliably imputed into our dataset. Remarkably, 8 of these 31 variants significantly affect 15 unique eQTL-genes (Q-Value < 0.05, Table 2).

**Table 2:** eQTL variants overlapping with genome-wide significant AMD variants.

IH*	dbSNP ID	CHR	Position [hg19]	Gene ID (ENSG)	Gene Symbol	P-Value	Q-Value	Effect Size**	SE	Non-risk allele	Risk allele	Frequency of risk allele	Distance to TSS
1.2	rs570618	chr1	196,657,064	ENSG00000244414	CFHR1	2.15E-12	4.34E-10	0.711	0.099	G	T	0.360307	−131822
1.1	rs10922109	chr1	196,704,632	ENSG00000134365	CFHR4	3.29E-24	1.66E-21	1.118	0.105	A	C	0.554124	−114738
1.1	rs10922109	chr1	196,704,632	ENSG00000244414	CFHR1	7.56E-24	2.54E-21	0.992	0.094	A	C	0.554124	−84254
1.1	rs10922109	chr1	196,704,632	ENSG00000116785	CFHR3	8.38E-17	2.11E-14	0.923	0.107	A	C	0.554124	−39292
1.1	rs10922109	chr1	196,704,632	ENSG00000143278	F13B	0.0002	0.012	0.216	0.057	A	C	0.554124	−303688
1.1	rs10922109	chr1	196,704,632	ENSG00000000971	CFH	0.0004	0.025	0.338	0.095	A	C	0.554124	83625
1.6	rs61818925	chr1	196,815,450	ENSG00000116785	CFHR3	1.38E-08	1.55E-06	0.649	0.113	G	T	0.417647	71526
1.6	rs61818925	chr1	196,815,450	ENSG00000244414	CFHR1	5.97E-05	0.006	0.416	0.103	G	T	0.417647	26564
1.6	rs61818925	chr1	196,815,450	ENSG00000134389	CFHR5	0.0001	0.011	−0.371	0.096	G	T	0.417647	−131216
11	rs7803454	chr7	99,991,548	ENSG00000121716	PILRB	5.67E-27	5.72E-24	0.251	0.022	C	T	0.188567	57812
11	rs7803454	chr7	99,991,548	ENSG00000085514	PILRA	6.16E-11	1.04E-08	0.372	0.056	C	T	0.188567	26396
23.1	rs2043085	chr15	58,680,954	ENSG00000128918	ALDH1A2	0.0002	0.016	0.207	0.056	T	C	0.667257	435333
23.2	rs2070895	chr15	58,723,939	ENSG00000166035	LIPC	5.45E-09	6.88E-07	0.561	0.095	A	G	0.80531	21172
23.2	rs2070895	chr15	58,723,939	ENSG00000137845	ADAM10	0.0003	0.021	−0.217	0.06	A	G	0.80531	−163463
24.2	rs17231506	chr16	56,994,528	ENSG00000087237	CETP	8.48E-05	0.008	−0.216	0.055	C	T	0.327434	−1233
27	rs6565597	chr17	79,526,821	ENSG00000182612	TSPAN10	1.70E-09	2.46E-07	−0.526	0.086	C	T	0.383459	−77375
27	rs6565597	chr17	79,526,821	ENSG00000184009	ACTG1	0.0002	0.016	0.312	0.084	C	T	0.383459	49825
27	rs6565597	chr17	79,526,821	ENSG00000141552	ANAPC11	0.0006	0.036	−0.171	0.05	C	T	0.383459	−321844

CHR: chromosome; TSS: transcription start site; SE: standard error of the effect size.

*IH: independent hit according to Fritsche *et al*.^3^.

**Effect size (beta) of a single AMD risk increasing allele.

Within the complement factor H (*CFH*) locus, several AMD associated variants appear to influence expression of *CFH* and CFH related genes (*CFHR*). Particularly, the independent hit variant rs10922109 (independent hit 1–1 in^3^) tags a common deletion of *CFHR1/CFHR3*. Since the deletion of both genes is protective against AMD, the risk increasing allele results in elevated expression of the two genes (Table 2).

Notably, two genes involved in HDL metabolism, Cholesteryl ester transfer protein (*CETP*) and hepatic lipase (*LIPC*), were both significantly regulated by AMD associated variants (Table 2). Specifically, rs17231506 is highly correlated to rs3764261 (R² > 0.99), a variant that results in markedly increased HDL levels in blood^31^. According to our eQTL data, rs17231506 reduces the expression of *CETP*, in line with the observation that *CETP* deficiency or pharmacological inhibition leads to elevated serum HDL. Further, our eQTL data showed that rs2070895 (−250 G > A) increases the expression of LIPC and would be expected to be associated with decreased HDL blood^32^.

Finally, we identified additional AMD associated variants that potentially act as eQTL in liver. The AMD risk increasing allele of rs7803454 increases the expression of the paired immunoglobin like type 2 receptor alpha (*PILRA*) and beta (*PILRB*) genes. The resulting proteins are known to function as antagonists within the Tyrosine-protein phosphatase non-receptor type 6 (PTPN6) pathway^33^ and have been implicated in both, AMD and Alzheimer’s disease risk^34^. Interestingly, we did not detect any eQTL within the strongest AMD associated locus located on chromosome 10q26 (*ARMS2*/*HTRA1*).

## Discussion

In this study, we have combined the genotypes and expression data of four previously published independent studies to further our understanding of the regulatory networks in liver tissue. Each individual study intended to identify new liver specific eQTL in order to elucidate the contribution of regulatory mechanisms on different diseases or traits. For example, Schadt *et al*.^18^ were the first to explore eQTL in liver tissue and correlated their results to genome-wide association studies of seven different diseases. AMD was not among them. Innocenti *et al*.^17^ and Schroeder *et al*.^19^ followed a similar approach but concentrated on the reproducibility of eQTL, while the latter group additionally focused on genes involved in drug response pathways. GTEx analysed eQTL in 44 human tissues and aimed to explore the interplay of gene regulation across tissues. By merging these resources this is to our knowledge the largest study on liver eQTL to date and promises to provide novel insight into the role of genetic variation on gene expression in liver tissue. Combining several studies while jointly analysing the data has drastically increased the power to detect novel eQTL across the genome. The replication rates of eQTL detected in individual studies can be as low as 38.5% (Table 1), even with a stringent FDR threshold of 0.1%. An approach known as mega-analysis has further improved the power of our study to detect novel eQTL. This also revealed a higher replication rate of eQTL identified by individual studies. Although the gain in power attributable to a mega-analysis can depend on the type of study^23^, the mega-analysis approach allowed us to identify additional, independent signals in 5% of the significant eQTL.

Mapping identified eQTL-variants against known gene structures such as introns, coding or non-coding exons revealed that a large proportion of the identified eQTL variants is highly enriched in intronic and coding regions of genes, in line with previous results^13,16^, although such an enrichment may be specific for certain tissues^35^. Similarly, we have observed a strong enrichment of eQTL variants in RegulomeDB classes one to four representing known eQTL and expected regulatory variants. Since many eQTL are shared between tissues^20^, an enrichment in RegulomeDB class 1 (representing known eQTL) is not surprising. Nevertheless, we also observe a strong enrichment of eQTL variants in RegulomeDB classes two to four, representing variants in experimentally determined regulatory epigenetic elements. Importantly, hypothetic regulatory variants in RegulomeDB class 5 (characterized by either transcription factor binding or a peak of DNase hypersensitivity) are not enriched in the identified liver eQTL variants, greatly increasing confidence in the robustness of our results. Alternatively, variants in RegulomeDB class 5 could be variants with weaker regulatory effects and thus, our study might be underpowered to identify significant eQTL variants that are characterized by mapping to a weak epigenetic mark.

Strikingly, the observed enrichment in gene structures were more pronounced in the independent hits which represented the most significantly associated variants and, in addition, the most significantly associated secondary signals. This strengthen the notion that the variant showing the smallest P-value of association or correlation in a locus is *a priori* the most likely one to be the true causative mutation^36^. Alternatively, it is also possible that the functional allele of the variant with the smallest P-value is rather tagging several haplotypes that affect gene expression in the same orientation^37^. Therefore, in case a defined eQTL is of major interest, such a locus has to be dissected further by statistical means to identify all independent haplotypes carrying functional alleles^10^.

While the central nervous system and the retina are expressing complement genes, the liver is nevertheless the primary site of synthesis for circulatory complement proteins^38^. In addition, the liver plays a key role in lipid metabolism^39^, besides the complement cascade another pathway implicated in AMD pathology by epidemiological and genetic studies. We therefore investigated whether any of the top hits of a recent GWAS for AMD^3^ are regulatory variants influencing gene expression in liver.

One of the most significant association signals for AMD resides within the *CFH* locus on chromosome 1 and represents a compound signal of two protective haplotypes tagged by the protective allele of the top variant^37^. One protective haplotype harbors a common deletion of the CFH-related genes 1 and 3 (*CFHR1/3*)^40^. The heterozygous deletion of both genes results in reduced levels of CFHR1/3 proteins in serum, while a homozygous deletion results in a complete absence of CFHR1/3^41,42^. In line with this, we found that the AMD risk increasing allele of rs6677604 is correlated to increased expression of both genes while the protective allele of rs6677604 (in strong linkage disequilibrium with the *CFHR1/3* deletion) is correlated with decreased expression. In addition, the protective allele reduces the expression of other *CFHR* genes as well as the expression of the *CFH* gene. Since CFH and CFH-related genes share high sequence identity with each other, the expression values of the individual gene may not be distinguishable from the related gene by currently used high-throughput methods^43–45^. Indeed, we found that the gene expression values of *CFH* and *CFH*-related genes (CFHR1-5) are correlated in liver samples (R² between 0.1 and 0.5).

One important result of our study reveals that two AMD-associated signals near *LIPC* and *CETP* are significant eQTL, strongly implicating HDL metabolism and serum lipid levels in AMD pathogenesis. We observed that the AMD risk increasing allele of rs17231506 reduces *CETP* expression, likely resulting in elevated HDL levels in serum^46^. This is in line with the observation that HDL levels are elevated in AMD patients compared to controls^7–9^. Further, the risk increasing allele of rs2070895 near LIPC results in increased expression of *LIPC*, which is generally associated with reduced serum HDL levels^47^. A study by Burgess and Smith^48^ also observed an AMD associated variant next to LIPC (rs261342) to be associated with decreased HDL serum levels^48^. This variant is in high linkage disequilibrium with rs2070895 (R^2^ = 0.84) which was shown in our study to cause elevated LIPC expression in liver. Burgess and Smith^48^ in addition demonstrated that the AMD risk associated variant rs261342 predominately results in reduced LDL and increased HDL levels. Of note, CETP and LIPC genes are key regulators of HDL remodelling which might be essential for efficient delivery of lipids (e.g. fatty acids, carotenoids) into the retina and efflux of excess lipids out of the retina. Importantly, CETP and LIPC variants have been shown to have additive effects on cardiovascular risk with low CETP activity variants combined with low LIPC activity variants increased the risk^49^. Cardiovascular risk could therefore add additional pressure to select specific variant gene combinations in the aged AMD population that were protected from cardiovascular death. A similar line of thought emerged from another recent study, which found that a genetic score based on genome-wide significant variants for elevated HDL serum levels was higher in AMD patients, strongly suggesting that AMD patients have more alleles that increase HDL than controls^50^, in line with other studies^51,52^. Other confounding variables such as exercise, drugs or alcohol consumption or the occurrence of AMD in study participants are potentially influencing our eQTL analysis. However, the individuals in the study were largely below 60 years of age (404 out of 588) and thus AMD associated impairment such as an overly sedentary life style should play a minor role in confounding our analysis. Furthermore, this study included a diverse and large set of individuals across multiple studies, which should reduce the effect of confounding environmental factors, especially since AMD associated factors are not likely to significantly influence confounders such as alcohol consumption^53,54^ or treatment with different, liver-metabolized drugs.

## Conclusions

We present the currently most comprehensive eQTL analysis for liver tissue and report that 1,959 out of 24,123 investigated genes have at least one significant eQTL in liver. Significant eQTL variants are more frequently found within gene boundaries and are more enriched in RegulomeDB classes representing likely regulatory variants. Several of these liver eQTL overlap with genetic variants strongly associated with AMD at genome-wide significance. These findings underscore the validity of the eQTL approach to identify disease-associated functional variants and provide further confirmation that HDL metabolism is strongly involved in AMD aetiology. Nevertheless, it should be emphasized that further replication of our results in disease relevant tissues such as retina or RPE or other functional validation studies are warranted. Specifically, this could further validate our notion that HDL metabolism is, in addition to the complement cascade, a major pathway in AMD disease development.

## Methods

### Genotype data

The genotypes of the four studies were retrieved from the respective databases (Table 1). Genotype quality control was performed for each study separately and, in addition, jointly after imputation. Since some studies reported only the zygosity of their samples at each variant (e.g. homozygosity: AA or BB; heterozygosity: AB), we first matched the reported alleles of each variant to the respective allele in the 1000 Genomes reference dataset to the Biomart^30,55^ online database (http://grch37.ensembl.org/biomart/). Multi-allelic variants were excluded to avoid potential ambiguity. Next, for each study we extracted the genotypes of all samples at 30,000 randomly chosen variants from all autosomes. We also included the genotypes of all samples from the 1000 Genomes Project Phase 3 (release 20130502)^56^ at the same variants and performed a PCA with the *snpgdsPCA* function of the *SNPRelate*^57^ package in R^58^. Since the haplotype structure can greatly vary between populations, we only included individuals clustering next to the European (EUR) reference individuals in the eQTL analyses (Supplementary Fig. S1). We then compared the reference allele in the datasets to the reference allele in the European 1000 Genomes samples. Alleles were flipped when given on the opposite strand. We excluded variants whose reference allele frequency differed by more than 10% from the reference allele frequency of the 1000 Genomes European samples. Furthermore, we excluded variants that were (1) not on autosomes, (2) had a minor allele frequency of MAF < 0.05 or deviated significantly from Hardy-Weinberg equilibrium^59^ (HWE, P < 1 × 10^−6^) after applying the respective function in the *VCFtools*^60^.

The individual genotype data sets were merged into a single VCF file. Variants which were not present in an individual study or were not genotyped in at least 100 samples were assigned missing in the respective individuals. Phasing and imputation was performed on the merged data, as accuracy of both algorithms increases with increasing sample sizes^61^. Phasing was performed with *SHAPEIT2* and standard settings by supplying the imputed genotypes from the 1000 Genomes Phase 3 reference panel^62^. The same reference panel was used to conduct a whole genome imputation with *IMPUTE2*^63^ at standard settings. Next, *VCFtools* was used to remove variants with a minor allele frequency < 5% and variants which showed evidence for a significant deviation from Hardy-Weinberg equilibrium (P < 1 × 10^−6^). In addition, variants with an IMPUTE2 info score smaller than 0.4 considered to be of low quality^64^, were removed. Finally, the reference allele frequency of each study was compared against the reference allele frequency of all other studies (Supplementary Fig. S2). Variants whose reference allele frequency differed by more than 15% between studies were excluded.

Specifics for each data set were as follows:

The *GTEx* data were retrieved through dbGAP^65^ (https://www.ncbi.nlm.nih.gov/gap, accession: phs000424.v6.p1). The positions of the variants were already reported based on the final hg19 build and thus, no additional lift-over was required.

Innocenti *et al*.^17^ genotype information was retrieved from the GEO database^66^ (accession code: GSE26105). The genotyping had been performed by the authors on an Illumina 610 Quad chip and the genotypes were encoded by each individual’s zygosity status (homozygosity: AA, BB; or heterozygosity: AB). The hg19 coordinates as well as the respective alleles of the variants were retrieved from Ensemble by querying the Biomart online database with the respective dbSNP identifier.

The genotype information from *Schroeder et al*.^19^ was retrieved from the GEO database (accession: GSE39036). The samples had been genotyped by the authors on an Illumina HumanHap300 chip and the genotypes were also encoded according to the individual zygosity status. The hg19 coordinates and alleles were retrieved from Ensemble as specified above.

The genotypes from the Schadt *et al*.^18^ study were retrieved from the Synapse database (accession: syn89614). The samples had been genotyped on either the Affymetrix 500k or the Illumina 650 Y genotyping chip. The genotype file included hg17 positions of each variant, a unique dbSNP identifier and both alleles of each individual. We initially removed variants without dbSNP identifiers and then used the program *liftover*^67^ from the UCSC Genome Browser (https://genome.ucsc.edu/util.html) to retrieve the hg19 coordinates of each variant.

### Gene expression data

The present study included the gene expression data from four independent studies. Three studies profiled gene expression by employing microarray platforms (Table 1) while one study used high-throughput transcriptome sequencing (RNA-Seq) for data generation. First, we remapped array probes to an *in silico* mRNA reference database based on Ensemble gene annotation^30^ with the help of the ReAnnotator pipeline^68^. Only exome-matching probes showing less than five mismatches were retained in the data set. Probes mapping to multiple genes or overlapping with common variants (according to dbSNP release 142) were removed from the analysis^69^. Probes which measured the gene expression of the same gene, were merged by calculating the mean of all probes within a gene, weighted by the variance of the respective probe over all samples. Hence, probes with a higher variance contributed more to the overall transcript levels than probes with little variation across samples.

For each data set, we performed basic expression normalization and quality control. Briefly, the available expression values were log2-transformed and a PCA was performed with the prcomp function in R to detect potential outlier samples within the dataset. We merged replicate samples by taking the mean of all replicate values.

The expression data of the four studies were merged and missing expression values were imputed using the *K-Nearest-Neighbour*^70^ method provided by the impute.knn function of the *impute* Bioconductor package^71^ in R. Genes that were included in one study but could not be imputed into the other studies were removed. Differences between all individuals were evaluated by conducting a PCA on the gene expression data (Supplementary Fig. S3A–C). In addition, the expression values for each individual were plotted as a boxplot (Supplementary Fig. S3D–F). Due to substantial differences between datasets, we applied further normalisation steps. Initially, we performed a quantile normalisation with the *normalize.quantiles* function of the R package *preprocessCore*^72,73^. Since quantile normalization alone was not sufficient to normalize all studies, we adopted an empirical batch correction method called ComBat with the *combat* function from the *sva* package in R^74^. By supplying known batch effects to the function (i.e. the study labels), ComBat standardises the data gene-wise and then applies an empirical batch effect correction (Supplementary Fig. S3C and F). The batch corrected expression values were used for the eQTL analyses, as no obvious bias of the single studies was noticeable.

Methods specific to the individual studies were as follows:

Firstly, for the GTEx data expression values (release GTex-V6p) were downloaded from the GTEx Portal (http://www.gtexportal.org/home/). The levels of transcript expression were encoded as “reads per kilobase of transcript per million mapped reads” (RPKM). We added 0.001 to all RPKM values to perform a log_2_ transformation of the data.

Secondly, the expression data from *Innocenti et al*.^17^ were retrieved from the gene expression omnibus (GEO) database (https://www.ncbi.nlm.nih.gov/geo/, accession: GSE25935). The expression values were already background subtracted and transformed to the log_2_ scale.

Thirdly, *Schadt et al*. (2008) provided a curated version of their data in the Synapse database (https://www.synapse.org/, accession: syn89614). As this study used an Agilent Custom 44k array, probe sequences were not openly available. In addition, not all samples had values for both genotype and gene expression data. The authors supplied an annotation file which links probe IDs to Ensemble and RefSeq^75^ identifiers. Expression values of probes were only used, if they were unanimously linked to a single Ensemble or RefSeq identifier. Furthermore, RefSeq identifiers were converted to Ensemble gene identifiers with the help of the Ensembl biomart tool^55^. A Shapiro–Wilk test^76^ revealed that raw values larger than 2 or smaller than −2 values are likely outliers. Thus, all of these were set to missing.

Finally, expression values from *Schroeder et al*.^19^ were retrieved from the GEO database (accession: GSE32504) as quantile normalized data. To retrieve probe sequences of the Illumina Human WG-6v2.0 chip for probe remapping, the *illuminaHumanv2.db* R package^77^ was used.

### eQTL analysis

Linear regression analysis between gene expression values and imputed allele dosages was performed with *Matrix eQTL*^78^. Age, gender and the first five principal components of the genotype PCA were included in the models as covariates. We exclusively calculated local eQTL (variant-gene distance less than one million base pairs) due to limited power to perform distant eQTL analyses^15^.

Two approaches were adopted to jointly analyse eQTL. First, a classic meta-analysis was applied to the individual study results. The effect size (slope) and standard error of the effect size were estimated with Matrix eQTL for each study separately. Further, a random effects model implemented in the function *MiMa*^79^ was applied to estimate the joint effect sizes and standard errors as well as the joint P-Values. The latter approach (mega-analysis) estimated local eQTL from the merged genotype and expression data directly. This approach also allowed us to search for novel independent eQTL for a gene by adjusting the linear regression model for the most significant eQTL variant for this gene. To account for multiple testing, the false discovery rate (FDR) was controlled to be smaller than 0.001. Thus, joint Q-Values were considered to be smaller than 0.001 for statistical significance.

### Functional annotation of eQTL variants

A control set of variants was generated by randomly choosing around 200,000 genetic variants within 1 Mbp of a gene locus (defined by the transcription start and stop site of each gene). A RegulomeDB score (www.regulomedb.org/*)* was then assigned to each control and eQTL variant. The score denotes the confidence that a certain variant is important for transcription factor binding or chromatin accessibility and thus gene regulation. Variants in classes one to four are deemed very likely regulatory variants, while variants in classes five to seven are less likely to influence gene expression. In addition, the Ensembl Variant Effect Predictor (*VEP*, www.ensembl.org/vep) was used to assign each eQTL variant to a functional consequence relative to known gene structures. The program predicted the most severe consequence per gene within a range of 1 Mbp up and downstream of each variant. For eQTL variants, only predicted consequences affecting the associated eQTL gene were evaluated. For the control variants, a single random consequence for a nearby gene was chosen.

### Ethics approval and consent to participate

This study used data of four public datasets. For further specifics on the respective ethics approvals, we refer to the single study publications.

### Data availability statement

All data are available in public databases as detailed in the methods section.

## Electronic supplementary material

Supplementary Data

Supplementary Table S1

Supplementary Table S2
