# Supplementary material for: A mega-analysis of expression quantitative trait loci (eQTL) provides insight into the regulatory architecture of gene expression variation in liver
Source: Sci Rep. 2018 Apr 12;8:5865. doi: 10.1038/s41598-018-24219-z (PMC5897392; doi:10.1038/s41598-018-24219-z)
Supplement: Supplementary file 1 — Supplementary Data [file 41598_2018_24219_MOESM1_ESM.docx]

Supplementary Information

**A mega-analysis of expression quantitative trait loci (eQTL) provides insight into the regulatory architecture of gene expression variation in liver**

Tobias Strunz, Felix Grassmann, Javier Gayán, Satu Nahkuri, Debora Souza-Costa, Cyrille Maugeais, Sascha Fauser, Everson Nogoceke and Bernhard H. F. Weber

This supplementary information file contains

- Supplementary Tables S1 to S5
- Supplementary Figures S1 to S3 including figure legends

**Supplementary Table S1.** Statistically significant results of the mega-analysis including four publically available studies and 588 individuals (Q-Value < 1x10-3).

Please see attached file “Supplementary Table S1.xlsx” (31,844 KB)

**Supplementary Table S2**. Independent signals of a mega-analysis including four publically available studies and inclusion of secondary signals for each significant eQTL gene.

Please see attached file “Supplementary Table S2.xlsx” (402 KB)

**Supplementary Table S3.** eQTL variants and eQTL genes at varying FDR thresholds

| **FDR** | **Schadt et al. 2008** | **Schroeder et al. 2013** | **Innocenti et al. 2011** | **GTEx** | **Meta** | **Mega** |
| --- | --- | --- | --- | --- | --- | --- |
| **5.00E-02** | 73999 / 1592 | 165518 / 3453 | 122474 / 2635 | 54639 / 1983 | 222521 / 4811 | 444276 / 7612 |
| **1.00E-03** | **29546 / 363** | **71423 / 913** | **52565 / 670** | **19802 / 387** | **101148 / 1313** | **202489 / 1959** |
| **1.00E-05** | 15779 / 200 | 36893 / 493 | 27754 / 364 | 10398 / 151 | 55958 / 727 | 117392 / 1125 |
| **1.00E-10** | 5400 / 68 | 12626 / 198 | 10053 / 134 | 3857 / 49 | 21943 / 300 | 45948 / 508 |
| **1.00E-20** | 1301 / 21 | 2344 / 46 | 2073 / 37 | 22 / 3 | 8861 / 118 | 14908 / 174 |

FDR = False Discovery Rate threshold

Meta= Meta-analysis

Mega=Mega-analysis

**Supplementary Table S4.** RegulomeDB scores of eQTL variants

| **RegulomeDB Score** | **RegulomeDB class annotation** | **Control** | **Control [%]** | **Mega** | **Mega [%]** | **IH** | **IH %** | **Fisher's exact test P-Value** | | |
| --- | --- | --- | --- | --- | --- | --- | --- | --- | --- | --- |
|  |  |  |  |  |  |  |  | **Control vs. Mega** | **Control vs. IH** | **Mega vs. IH** |
| 1 | Known eQTL in other tissue | 1422 | 0.702 | 9488 | 5.160 | 175 | 8.578 | < 1.00E-150 | 2.33E-122 | 8.27E-11 |
| 2 | TF binding + DNAse peak + TF motif | 4224 | 2.086 | 5438 | 2.957 | 110 | 5.392 | 4.40E-67 | 1.61E-18 | 3.43E-09 |
| 3 | TF binding + DNAse peak + any motif | 5009 | 2.474 | 5098 | 2.773 | 86 | 4.216 | 6.82E-09 | 2.67E-06 | 0.0001727 |
| 4 | TF binding + DNAse peak | 12070 | 5.961 | 13982 | 7.604 | 222 | 10.882 | 8.25E-92 | 1.11E-17 | 7.87E-08 |
| 5 | TF binding or DNase peak | 44074 | 21.766 | 40069 | 21.792 | 448 | 21.961 | 0.879 | 0.664 | 0.684 |
| 6 | Motif only | 64712 | 31.958 | 56374 | 30.659 | 555 | 27.206 | 1.59E-18 | 1.15E-05 | 0.0018067 |
| 7 | No annotation | 70978 | 35.053 | 53423 | 29.054 | 444 | 21.765 | < 1.00E-150 | 1.47E-44 | 5.88E-17 |

Mega: mega-analysis variants; IH: independent hit variants; Control: 202,489 randomly chosen variants within 1,000,000 bases of a gene

| **Classification** | **Control** | **Control %** | **Mega** | **Mega %** | **IH** | **IH %** | **Fisher's exact test P-Value** | | |
| --- | --- | --- | --- | --- | --- | --- | --- | --- | --- |
|  |  |  |  |  |  |  | **Control vs. Mega** | **Control vs. IH** | **Mega vs. IH** |
| Upstream gene | 99664 | 49.219 | 78606 | 42.750 | 779 | 38.186 | < 1.00E-150 | 5.97E-28 | 1.79E-07 |
| Downstream gene | 99406 | 49.092 | 68537 | 37.274 | 660 | 32.353 | < 1.00E-150 | 5.24E-63 | 2.62E-10 |
| Intron | 3295 | 1.627 | 33964 | 18.472 | 486 | 23.824 | < 1.00E-150 | < 1.00E-150 | 6.95E-17 |
| Non coding transcript exon | 60 | 0.030 | 265 | 0.144 | 13 | 0.637 | 8.42E-40 | 5.88E-14 | 5.58E-06 |
| 3 prime UTR | 36 | 0.018 | 1228 | 0.668 | 42 | 2.059 | < 1.00E-150 | 8.83E-66 | 3.21E-11 |
| 5 prime UTR | 3 | 0.001 | 375 | 0.204 | 27 | 1.324 | 2.78E-121 | 3.56E-53 | 1.53E-14 |
| Missense | 10 | 0.005 | 389 | 0.212 | 15 | 0.735 | 1.98E-115 | 2.32E-25 | 1.74E-05 |
| Synonymous | 10 | 0.005 | 361 | 0.196 | 5 | 0.245 | 2.80E-106 | 1.21E-07 | 0.421 |
| Splice site | 5 | 0.002 | 125 | 0.068 | 9 | 0.441 | 8.33E-36 | 4.20E-16 | 8.88E-06 |
| Loss of function | 0 | 0.000 | 22 | 0.012 | 4 | 0.196 | 3.73E-08 | 5.11E-09 | 0.001 |

**Supplementary Table S5.** Consequences of eQTL variants predicted by Ensembl Variant Effect Predictor (VEP)

Mega: mega-analysis variants; IH: independent hit variants; Control: 202,489 randomly chosen variants within 1,000,000 bases of a gene


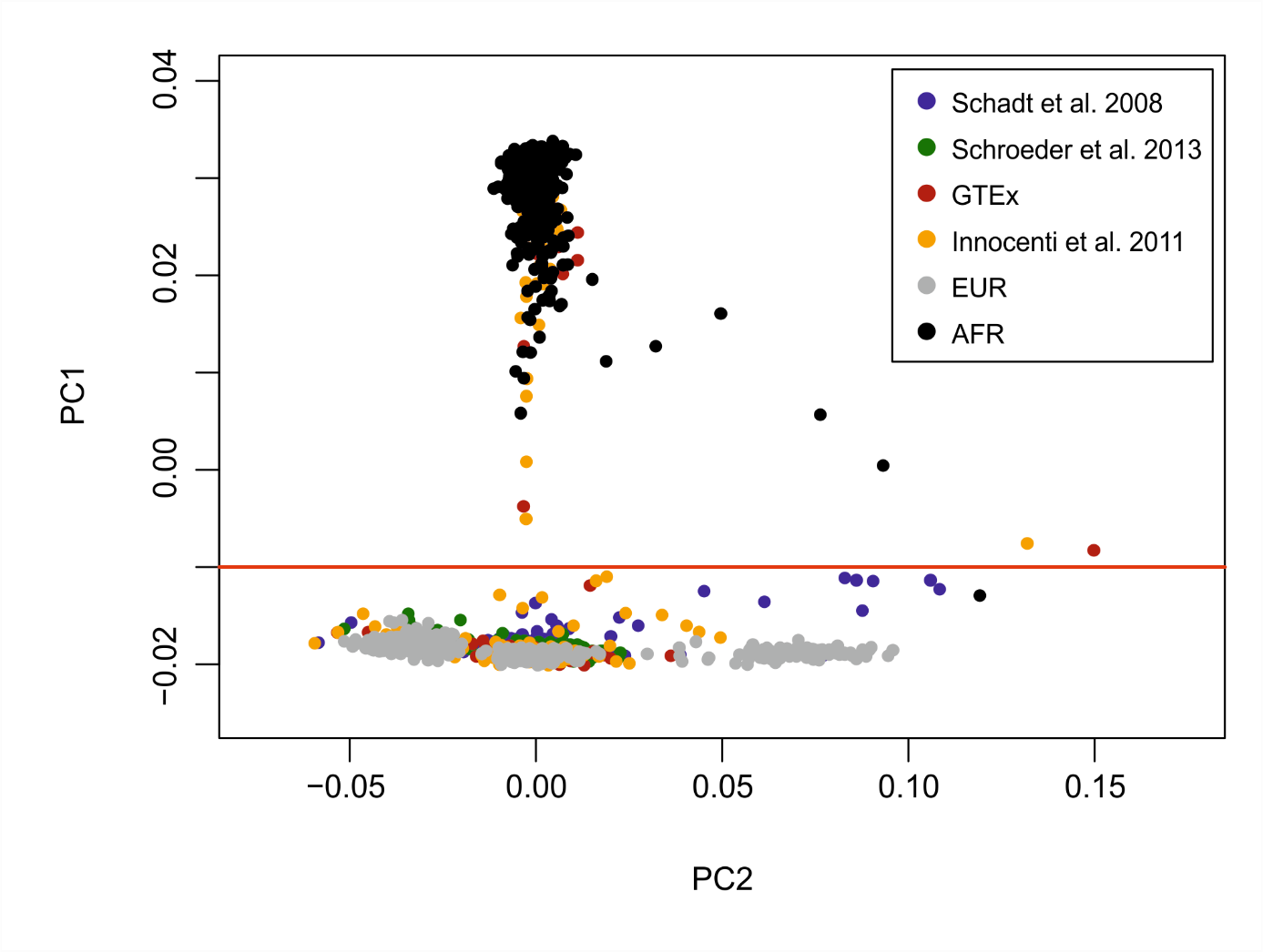


**Supplementary Figure S1.** Genotype Principal Component Analysis. 30,000 variants were chosen at random from the autosomes and the genotypes of those variants were extracted from the four datasets (GTEx, Innocenti et al., Schadt et al., and Schroeder et al.). In addition, we extracted the genotypes of samples of European (EUR) or African (AFR) ancestry from the 1000 Genomes Project and performed a Principal Component Analysis (PCA). The horizontal red line indicates the threshold for exclusion. Accordingly, individuals showing a principal component 1 value greater than -0.01 were excluded, as they were not deemed of European ancestry.


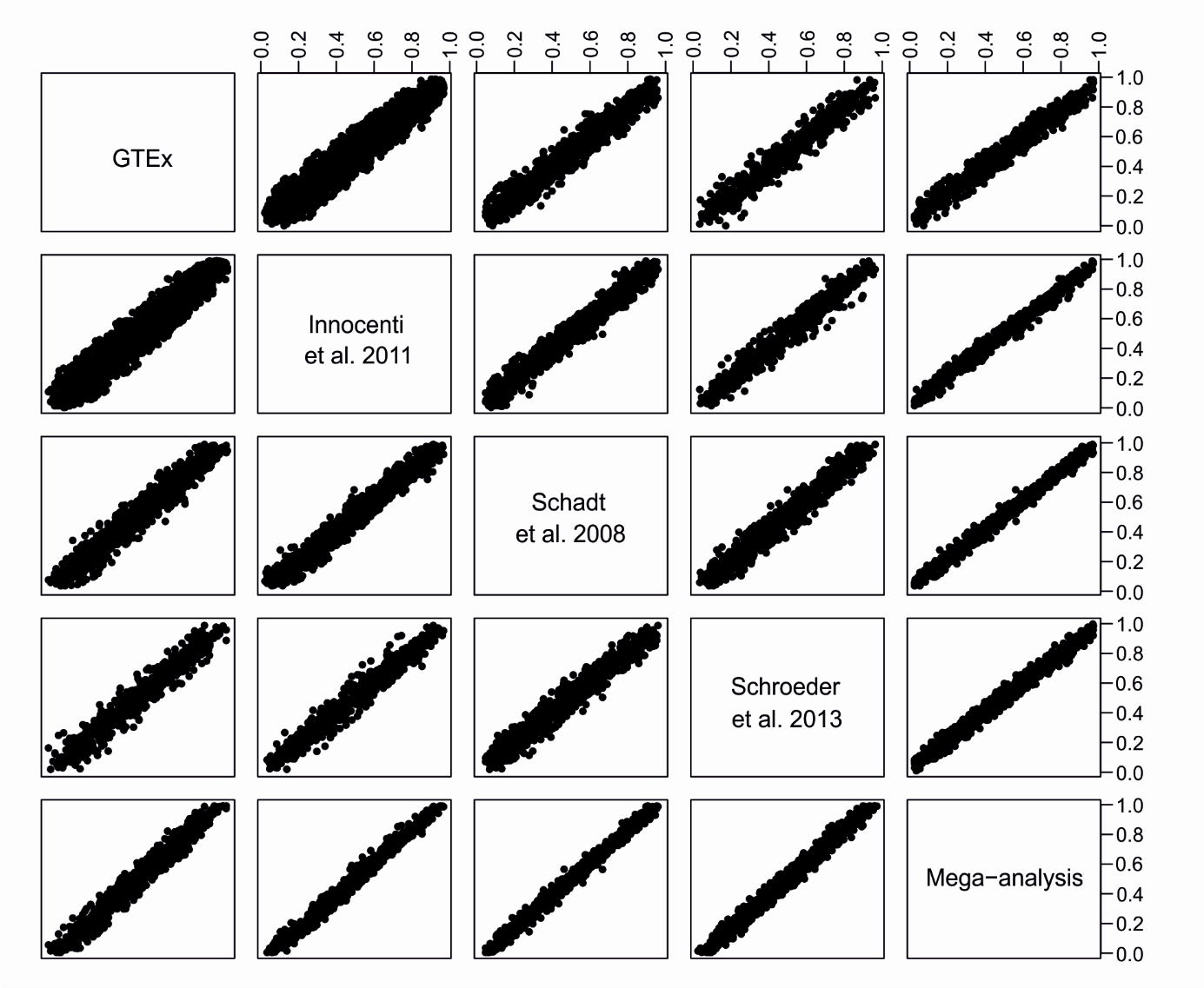


**Supplementary Figure S2.** Comparison of the frequency of all variants in the four individual datasets as well as in the combined dataset after imputation and quality control. The frequency of the reference (REF) alleles in each of the five datasets (GTEx, Innocenti et al., Schadt et al., Schroeder et al. and combined dataset termed “Mega-analysis”) was plotted against all other datasets.


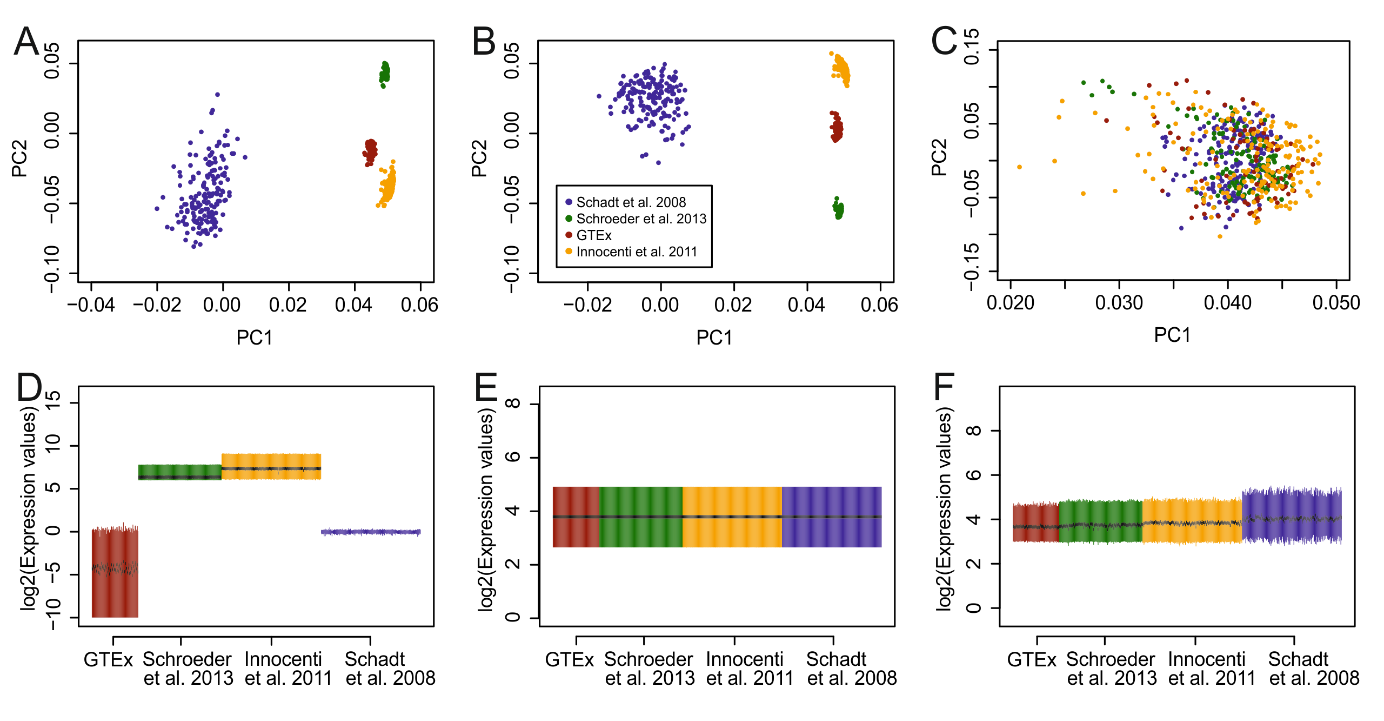


**Supplementary Figure S3.** Gene expression data normalization process. A PCA was conducted on the merged gene expression data of the four datasets (GTEx, Innocenti et al., Schadt et al., Schroeder et al), at three different consecutive normalization steps: **(A)** raw log_2_ transformed merged data (no normalization), **(B)** quantile normalized data and **(C)** after adjustment for known batch effects using ComBat ^69^. In addition, the gene expression values are presented as a boxplots at the same stages **(D-F)**.
